# Producing more effective physician leaders through medical training: Expanding the focus beyond the doctor-patient relationship

**DOI:** 10.1177/08404704251327091

**Published:** 2025-03-19

**Authors:** Mark Downing

**Affiliations:** 1 3688 Dalhousie University, Halifax, Nova Scotia, Canada.

## Abstract

Most of what physicians learn in their training when it comes to ethics focuses on the principles related to the doctor-patient relationship: beneficence, non-maleficence, and autonomy. At a system level, this translates into an obligation for physicians to advocate for their patients based on these principles. Advocacy does not necessarily have answers when resources are scarce, and as a result, physicians often find that they are not “at the table” when important decisions are made at the organizational level. I will argue that for physicians to be more effective leaders within their organizations, there needs to be more of a focus on principle of justice within medical training, specifically when it comes to theories around resource allocation and social justice. This will help physicians to more effectively advocate for their patients, have conversations with health leaders who have different points of view, and participate in organizational decision-making.

## Introduction

Physicians are often perceived as leaders within healthcare organizations, yet a common refrain from physicians is that organizational leadership does not listen to them and they don’t have “a seat at the table” when important decisions are being made. This was particularly true during the COVID-19 pandemic, when resources became scarce, and decision-making was often driven more by ethical considerations than by medical expertise. Physicians’ work has “long been organized around practice norms of independence and autonomy,” and even when physicians are employees of organizations, “their work is only loosely controlled by organizational leaders and managers.”^1^ This distance from organizational leadership allows physicians to be effective advocates for their patients, and in many ways health advocacy is the primary focus for physician leadership as a result. Yet health advocacy is often unable to provide a solution when a difficult decision needs to be made around a scarce resource where multiple patient populations have a reasonable claim, as was particularly true during the pandemic. This is why “the table” is often confined to hospital administrators, who are in the best position to think about the system as a whole and weigh the consequences of resource allocation decisions. In order for physicians to better participate in health leadership, medical education needs to reconsider how physicians can help their organizations to make difficult decisions when resources are scarce. This requires a more solid foundation in the principle of *justice* for medical trainees.

## Why professional identity formation in medical training creates leaders who focus on the individual

In Canada and throughout many countries around the world, the CanMEDS framework has served as a template for medical professional identity formation since the 1990s.^2^ Produced by the Royal College of Physicians and Surgeons of Canada (RCPSC), CanMEDS “identifies and describes the abilities physicians require to effectively meet the healthcare needs of the people they serve.” Physicians are primarily viewed as medical experts within this framework, a central competency that links six other key competencies: communicator, collaborator, leader, health advocate, scholar, and professional. The last iteration of CanMEDS is from 2015 and since then, there have been several important events that influenced how medicine is practiced, including the COVID-19 pandemic, worsening climate change, the Truth and Reconciliation Commission’s findings (specifically call to action number 24 highlighting the need for more medical training in Aboriginal health issues^3^), and the death of George Floyd sparking the Black Lives Matter movement. The next iteration of the CanMEDS framework is currently in development, and the RCPSC working group has acknowledged that what it means to be a physician is changing as a result of these events.^4^

Professional identity formation throughout the history of medicine has been largely guided by normative thinking based on a core group of ethical principles. The “classical” Hippocratic Oath^5^ is based primarily on the principles of beneficence and non-maleficence, and these have served and continue to serve as guiding principles since medicine was first established as a profession. Later in the 20^th^ century, the principle of autonomy became more of a central focus of medicine, such as through the Nuremberg Code in the context of medical research.^6^ These three principles primarily focus on the relationship between the physician and the patient and largely neglect how choices made within this relationship impact other patients, the healthcare system, and society in general.

### Adapting to a changing society: Why the medical profession needs to incorporate a broader understanding of justice

Justice is considered to be the fourth and final overarching principle in bioethics as first argued by Beauchamp and Childress in the 1970s^7^ and looks beyond individual relationships to questions about fairness. The current CanMEDS framework acknowledges that a physician has many roles outside of the physician-patient relationship, such as collaborator and health advocate, yet it does not provide much guidance in how a theoretical foundation in justice can help physicians to realize these roles. A RCPSC working group undertook a systematic review of the medical education literature in 2023 and identified eleven “emerging concepts” that are being used to explore how the next CanMEDS framework should change, and five of these themes have a strong focus on justice: anti-racism; equity, diversity, inclusivity, and social justice; complex adaptive systems (understanding the healthcare system); Indigenous health; and planetary health.^8^ The working group has acknowledged that the next version of CanMEDS cannot be business as usual: “Our understanding of health and illness has been fundamentally altered by critical events. This creates an imperative for rights-based change and action in medicine and medical education.”^4^ If the intention of the RCPSC is to shift towards something that better balances patient rights, then this is also a shift away from the traditional principles of beneficence, non-maleficence, and autonomy towards the principle of justice.

There is an increasing understanding, especially since the pandemic, that healthcare system resources are finite, and that increasingly clinicians need to consider impacts on others when making decisions for their patients. The current iteration of CanMEDS focuses on healthcare resource *stewardship* as a competency, yet stewardship is aimed at improving overall capacity in the system and does not necessarily provide physicians with the skills to make difficult decisions concerning which patients get what resources when they are limited. There is also a strong focus on *advocacy*, but again a lack of practical advice on how to do this effectively and appropriately when resources are limited. A focus on major theories of distributive justice (utilitarianism, egalitarianism, libertarianism, and communitarianism), procedural justice, and social justice would help to give physicians the critical reasoning skills to better address these problems. By better understanding what is considered fair, and how fairness can be conceived differently based on different theories of justice, physicians can advocate more effectively for their patients, as well as get a “seat at the table” where they can have constructive conversations with other health leaders who have different points of view.

Social justice theory, specifically, needs to be a focus for physicians-in-training going forward in considering the impacts of racism and colonization. Traditionally in medicine, patient rights have often been interchangeable with patient autonomy and a sense of individualism. This way of thinking fits best within theories of distributive justice, where fairness means treating like individuals the same and focusing on the resources that are owed to them. Yet Iris Marion Young points out that humans are primarily social beings, and the most important factor to consider around justice is therefore the power in our relationships, which is not a “thing” that adheres to the “logic of distribution.”^9^ In a social justice mindsight, domination and oppression become the primary concerns in addressing patient rights. Kannin Osei-Tutu argues for a transformative change in our approach to physician competencies: “Globally, health care systems grapple with deep-rooted health disparities perpetuated by injustice and biases. This demands that the physician’s responsibilities extend beyond medical expertise—which is the cornerstone of the profession, as it should be—to encompass principles of inclusive compassion and social justice, adapting to the evolving needs of society.”^10^ Several of the key social movements motivating the current discussions around CanMEDS (in particular anti-Black racism and Indigenous rights) focus on oppression as a root cause of injustice, and this is where theories of social justice will have answers when distributive justice does not.

## Next steps: Navigating the hidden curriculum in medical training to improve ethical reasoning when it comes to justice

A strong grounding in justice and resource allocation can also equip physicians to better approach the many small ethical dilemmas in their daily lives. Should I squeeze in another urgent patient appointment into my overbooked clinic? Should I delay that next consult and get lunch first? Medical school curriculums tend to focus on extreme dilemmas in order to draw substantial contrasts in teaching ethics, yet one downside to this approach is that trainees may not realize that they are constantly making many important ethical decisions implicitly on a daily basis. Furthermore, this can often leave bioethics (and bioethicists) siloed within the formal teaching curriculum, whereas most of what is taught in medical school is within the “hidden curriculum” that occurs in the clinical context. The hidden curriculum is more concerned with “replicating the culture of medicine than with the teaching of knowledge and techniques” and in fact may be “antithetical to the goals and content” of what is formally offered.^11^

“Microethics” seeks to shift the focus in ethical teaching from extreme dilemmas to everyday issues so that trainees can shape a better morality for themselves in the real world.^12^ Consider the art of “putting up a wall,” where every effort is made to redirect patient consultation requests to other services. This can be socialized through residency as a “good” way to survive a night of call. One way to address this would be to talk about “the wall” more formally in the medical school curriculum. This could be within a tutorial dedicated to medical triage. Instead of choosing something dramatic like a mass casualty scenario, perhaps groups could examine something more routine like how to triage consults as an internal medicine resident during a standard overnight call shift. Students would then talk explicitly about what “the wall” means, and what ethical foundations it has, which would land on a Libertarian way of thinking in prioritizing physician autonomy over the needs of patients, colleagues, or the organization. The flaws of this approach would then be discussed, specifically how it leads to gridlock in the emergency department, conflict amongst colleagues, and treating patients as a means to an end. A shift outside of distributive logic may provide the best solutions in the tutorial. If services cannot agree on a fair distribution of patients, then at least they may be able to agree on a fair process in allocating consults, and this is where a *procedural justice* approach can be of use, such as the Accountability for Reasonableness framework.^13^ Furthermore, from a social justice perspective, one might argue that the primary reason that patients are being perceived as burdens in academic institutions is due to the culture and practices that lead to overworked trainees; therefore, solutions should focus on supports for trainees so that avoiding burnout is not always front and centre during an overnight shift. Increasingly this approach is being adopted in medical education, where “fatigue risk management” has been an area of focus in creating a just culture learning environment.^14^ The goal of this exercise would be to better equip trainees to identify and mitigate gaps in their own moral thinking when it comes to overnight call coverage.

More justice theory in medical school also means more educators with expertise in justice theory. Yet beyond simply recruiting more ethicists, the approach discussed above emphasizes a need to understand how theory can be applied in practice, particularly within the everyday context. This first requires physician educators who can identify what gaps there are within the formal curriculum that are not covering ethical dilemmas concealed within the hidden curriculum. Ethicists are then in the best position to inform the theoretical foundations that drive the discussion around these cases and provide students with the tools they need to identify and address these dilemmas when they arise down the road in practice.

### Down the road from medical school: Producing better leaders with a focus on organizational ethics

As students step back from their direct patient interactions and apply frameworks based on theories of justice to their daily micro-ethical dilemmas, they will be better equipped to tackle the larger and more complex organizational issues down the road. One key skill for physicians to be successful along this path is the ability to put themselves in the hospital administrators’ shoes, and this is where thinking about different theoretical approaches can help, starting with basic theories of distributive justice (Table 1).

**Table 1.** Common theories of distributive justice and their applications within healthcare (adapted from Beauchamp and Childress^7^).

Theory	Description	Examples of application within healthcare
Utilitarianism	Maximize *social utility*. Often understood through a cost-benefit analysis. “The ends justify the means.”	Often an appropriate approach for larger organizations or communities (such as public health), or when in a crisis (such as mass casualty triage).
Libertarianism	Emphasis on liberty, property rights, and the free market. Aligns well with *procedural justice*, where unequal outcomes can be justified if a fair procedure is followed.	More reflective of the patient perspective as respect for autonomy has become a more prominent principle in medical practice.
Egalitarianism	Focus on *equality of*:	The justification for most publicly funded healthcare systems rests on egalitarian principles. Increasingly the medical profession is focused on egalitarian principles, focusing on health equity and social justice.
	Opportunity (e.g., access to healthcare)	
	Capability (e.g., social determinants of health)	
	Well-being (e.g., health)	
	More recent versions have focused on equity and social justice.	
Communitarianism	What is important to the *community as a whole* as opposed to *individuals* within the community. A focus on general welfare and common purpose.	An institution’s mission, vision, and values are based on communitarian considerations.

Consider a Department Head for Orthopedics who just finished a successful fundraising campaign to build a new unit for post-operative care, only to see the hospital convert this unit into a general medicine ward to accommodate the growing number of medicine patients waiting for beds in the Emergency Department. The Department Head is frustrated and is likely to view this situation through the lens of their patients and surgical colleagues, viewing this as unfair primarily because they should be able to enjoy the rewards of their own fundraising activities. At heart this is a *Libertarian* argument, where what’s owed is primarily based on property rights and the free market. Conversely, the administration has adopted a *Utilitarian* approach, where the “ends justify the means,” and in this case that is maximizing inpatient bed capacity to free up space in the Emergency Department. Thinking about these labels helps to establish that both positions can be reasonable, rather than simply arguing that the “right” position is what is best for “my patients” or “the system.” This will help to foster better dialogue and empathy at the leadership table.

In this case, *Communitarianism* can offer a third perspective to help both sides reach a consensus. Here, the lens shifts from groups of individuals to thinking about the community as a whole, and what decisions are most in keeping with that community’s values. It may be challenging to pin down what exactly those values are (or who gets to decide), but a starting place would be to consult with the hospital foundation, who may have good relationships with both the Department Head and the hospital administration and is also well-situated to understand whether the community would support a rapid switch in the utilization of resources it helped to procure. Further work to engage with the community directly, applying principles from procedural and social justice, would help to more clearly understand what the “right” decision is here. Ultimately, this is not about what the department or the hospital wants or needs, but rather how they can best serve their community together.

## Conclusion

The social context that we live in is changing and what it means to be a physician is changing as a result. Most of what a physician brings to the table within healthcare organizations is medical expertise and the ethical perspective that comes from the patient-doctor relationship. To be effective leaders within their organizations, physicians need to incorporate these assets within a broader thinking about justice, particularly when it comes to resource allocation and social justice. Focusing more explicitly on this early in medical training will help to produce better physician leaders in the future.
